# Conceptual Framework for Smart Health: A Multi-Dimensional Model Using IPO Logic to Link Drivers and Outcomes

**DOI:** 10.3390/ijerph192416742

**Published:** 2022-12-13

**Authors:** Jianwei Deng, Sibo Huang, Liuan Wang, Wenhao Deng, Tianan Yang

**Affiliations:** 1 School of Management and Economics, Beijing Institute of Technology, Beijing 100081, China; 2 Sustainable Development Research Institute for Economy and Society of Beijing, Beijing 100081, China

**Keywords:** smart health, Delphi process, IPO model, sustainable development

## Abstract

Smart health is considered to be a new phase in the application of information and communication technologies (ICT) in healthcare that can improve its efficiency and sustainability. However, based on our literature review on the concept of smart health, there is a lack of a comprehensive perspective on the concept of smart health and a framework for how to link the drivers and outcomes of smart health. This paper aims to interweave the drivers and outcomes in a multi-dimensional framework under the input–process–output (IPO) logic of the “system view” so as to promote a deeper understanding of the model of smart health. In addition to the collection of studies, we used the modified Delphi method (MDM) to invite 10 experts from different fields, and the views of the panelists were analyzed and integrated through a three-round iterative process to reach a consensus on the elements included in the conceptual framework. The study revealed that smart health contains five drivers (community, technology, policy, service, and management) and eight outcomes (efficient, smart, sustainable, planned, trustworthy, safe, equitable, health-beneficial, and economic). They all represent a unique aspect of smart health. This paper expands the research horizon of smart health, shifting from a single technology to multiple perspectives, such as community and management, to guide the development of policies and plans in order to promote smart health.

## 1. Introduction

The last two decades have been called the golden age of global health [1]. As an expensive but essential public service, the demand for healthcare is increasing alongside economic growth. People have come to recognize that a comprehensive and sustainable health system not only brings significant benefits to the physical and mental health of individuals but also provides the driving force for harmonious, stable, and orderly economic and social development [2]. However, the current difficulties in accessing healthcare and its high costs have seriously constrained the development of medical care in both developed and developing countries lacking a public health system that promotes social justice and equity, constituting a dilemma that has mainly been caused by an uneven distribution of medical resources, the poor equity of and accessibility to healthcare services, and high healthcare costs. Smart health can be an effective solution to this problem. Smart health makes healthcare systems more accessible and personalized in order to effectively improve the health of citizens and increase the effectiveness of health-related services to reduce healthcare expenditures and increase efficiency [3]. The massive market demand in the smart health industry has great potential for growth.

Although smart health is in a golden age of rapid development, a clear and unified definition of smart health is still lacking [4], and we still face multiple challenges with respect to achieving the ideal vision of smart health. We have summarized the most popular definitions from the current literature and attempted to segregate the key themes of smart health from these definitions in Appendix A. Almost all the definitions show a positive impact. Many scholars have discussed the impact of the IoT [5], AI [6], wearable devices [7], and big data [8] on the development of smart health from the perspective of technology. No fewer than 50 smart medical monitoring systems have been invented [9]. While the technology of smart health continues to evolve, there is no denying that the development of smart health technology is still in its infancy, and there are many barriers before it can be widely implemented [10]. However, the current discourse on smart projects is overly focused on the capabilities and development of technology [11], which is a common problem in the reform of the current health system. Many technologies are not successful enough in achieving sustainable innovation in healthcare practice, which may be because the development of medical technology is driven by experts; consequently, the technology is unable to meet the needs of users and ignores social and cultural habits. Scholars have criticized the narrow pursuit of technological innovation as serving the benefits of specific enterprises, but have not considered disruptive social problems and the specific goal of improving the livelihoods of residents [12]. In fact, especially in the field of health, the more technologies that adapt to humans and organizations, the greater the potential of the system [13]. Most of the existing evaluation studies express concern about technical issues or clinical processes, but these do not explain why such issues/processes have negative effects on specific users in specific environments [14,15]. In addition to technology, some scholars have attempted to determine the implications of smart health from the perspective of management and service. Smart health has greater environmental [16] and economic impacts [17] and has a significant optimization-related effect on resource allocation, thus greatly facilitating the management of hospitals. Simultaneously, smart health integrates patients and doctors into a common platform for smart health monitoring by analyzing daily human activities [18]. Furthermore, smart health enables patients and doctors to communicate with each other through a mobile platform, while also enabling cross-regional and cross-country consultations, which demonstrates the true realization of mobile healthcare and telemedicine and, consequently, improves service quality.

As an important part of healthcare 4.0 [19], the goal of smart health should be consistent with the health system in question. The Committee on Quality Health Care in America of the Institute of Medicine (IOM) states that health systems in the 21st century should strive to improve six dimensions of quality of care: safety, effectiveness, patient-centeredness, timeliness, efficiency, and equity [20]. International organizations, including the World Health Organization (WHO), and multinational health system evaluations have relied on this research, on whose basis the Lancet Global Health Commission defines a high-quality health system [21] as follows: “A high-quality health system is one that optimizes health care in a given context by consistently delivering care that improves or maintains health outcomes, by being valued and trusted by all people, and by responding to changing population needs”. A quality health system is based on four values: its primary concern is its patients and it is equitable, resilient, and efficient.

In this context, it is necessary to better understand the implications, drivers, and outcomes of smart health in order to better define a new concept. IPO is defined as “inputs that lead to processes that in turn lead to outcomes” [22], which can help designers evaluate and improve designs and correct previous implementation flaws [23]. This study applies the IPO model to link three variables, namely, the health system, drivers, and outcomes, to present a more comprehensive process and causal model of smart health. Under the IPO logic, the input variable is the “health system” itself, which can also serve as an “asset” of smart health, and the outcome is the output variable, which also serves as the “output” of smart health. The process variables are the “drivers” or the “processes” of smart health. The internal logic of the framework is that there is a chain of cause–effect linkages, which starts from the drivers, moves through to the expected outcomes, and progresses to the sustainable development of smart health. Considering the effectiveness and efficiency of the IPO model, it ultimately transforms the health system (“input”) into smart health (“output”).

**Figure 1:**
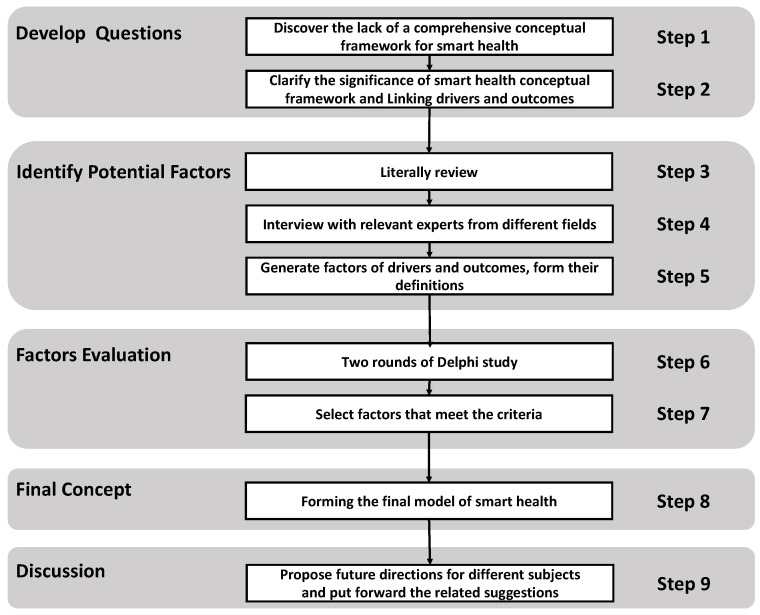
Process of conceptual development of smart health.

We developed a multidimensional conceptualization framework that incorporates the drivers and outcomes of smart health. We expect this systematic review to provide a direction for researchers, policymakers, and all smart health practitioners. Figure 1 shows the methodology of conceptual development.

## 2. Methods

### 2.1. Modified Delphi Method

The Delphi method [24] was originally developed by RAND for a certain topic. In this method, after one or more rounds of collecting and adopting different responses and opinions from relevant experts, experts can change their opinions after each round and finally synthesize the statistical results of the feedback until a consensus is reached on the set criteria. However, this also reveals some shortcomings, such as the need for repeated surveys to reach a consensus among experts, which can be considerably time consuming and hamper the completion of a given study [25]. As an alternative to the traditional Delphi method, the modified Delphi method (MDM) was created [26]. It not only has the inherent advantages of the traditional Delphi method but also effectively reduces the impact of bias caused by group interactions.

Referring to Wu and Chen’s [27] given paradigm, the steps of the MDM are as follows:

(1) Review the literature and develop a questionnaire;
(2) Form a group of experts;
(3) Distribute the questionnaire on expert opinions;
(4) Analyze and integrate group opinions;
(5) Conduct a second round of questionnaire design and surveys;
(6) Achieve a consensus.

**Figure 2:**
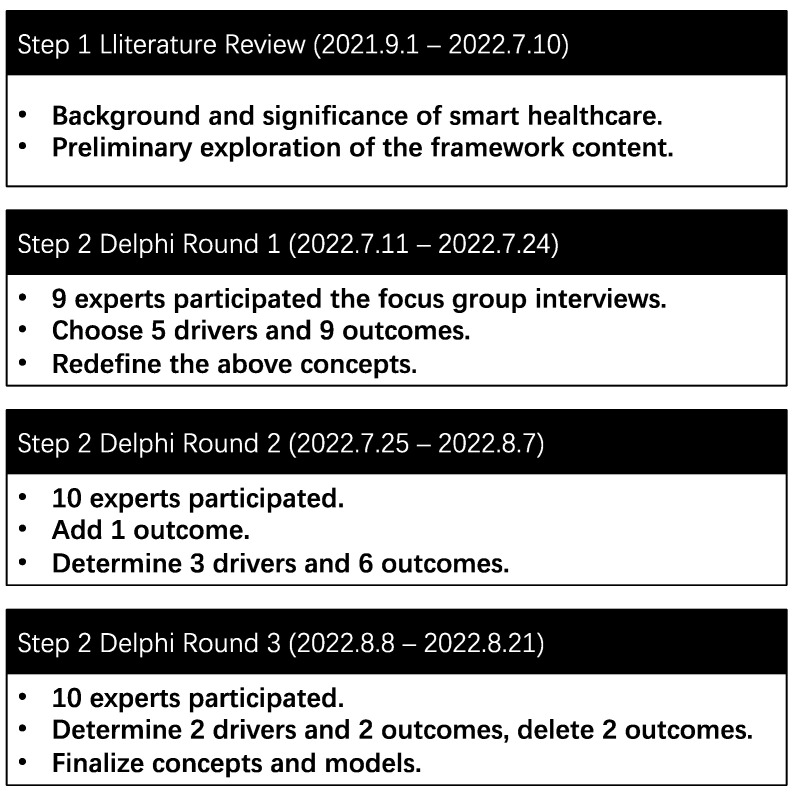
Overall process of factor development for smart health.

Figure 2 shows the flow chart for this study.

### 2.2. Literature Review

According to the framework report of the category review [28], the peer-reviewed literature was analyzed to capture the definition of smart health as accurately as possible. We searched the literature published from November 2008 (the first introduction of the concept of smart health by IBM) to July 2022, including databases such as Web of Science and PubMed.

Preliminary searches indicated that the volume of research appeared sparse. To be included in this article, studies were assessed against the following criteria:

(1) The study must constitute primary research of any design;
(2) It must be published in print format or on the Internet;
(3) It must contain a definition or attempt to define smart health in clear terms;
(4) It must be relevant to health or health systems.

The research team agreed upon a list of terms that were used to identify potentially relevant studies. The search scope included common fields, such as title, abstract, and keywords, and the keywords included “smart health”, “intelligent health”, “smart medical”, “intelligent medical”, and “health system.”

The study selection process was completed in two stages. Titles and abstracts were independently screened against the inclusion criteria by two reviewers (JD and SH). Studies appearing to meet the criteria were obtained as full text articles, which were independently screened using the same criteria (JD, SH, and TY). Any differences or disagreements were resolved through discussion with the whole team. The references of the reviewed articles were also searched for additional relevant sources, and duplicate definitions were removed.

### 2.3. Focus Group Interviews

The first round of consultation was designed to elicit broad and general concepts from the panelists through unstructured, open-ended questions. Focus group interviews were organized to gather information from different participants’ perspectives to provide researchers with the necessary data [29], which were used extensively for research on poorly understood or ill-defined topics [30]. The number of focus group interviews should not be too large, and each group’s context should not be overly broad; otherwise, there may be a tendency to divide opinions within the group [29]. We collected feedback from some of the researchers and scholars in the first round.

Subsequently, the participants held three discussions, and an outline of the interviews was developed based on the literature review and discussions within the research team, which included the following questions:

(1) In your opinion, what is the purpose of smart health?
(2) What are the three to five characteristics of smart health?
(3) How can smart health be achieved?
(4) What is the current impact of smart health on personal and professional healthcare?
(5) What impact is smart health likely to have in the next 5 years?

During the interview process, a moderator and a notetaker were assigned to record participants’ opinions, mood changes, and nonverbal behaviors. The moderator used open-ended questions to guide the questions without expressing personal opinions, and the interviews were used to gather the views of different scholars on smart health.

### 2.4. Factor Generation

The five drivers and nine expected outcomes for inclusion in the discussion were identified through the first round of focus-group interviews in conjunction with the literature review. We listed each opt-in factor, combined with definitions from the literature, and modified the details by considering the characteristics of smart health to generate definitions for each concept and use them for expert consultation (Table 1).

### 2.5. Profile of Panel

In accordance with existing research, the selected panelists were required to have experience working in a relevant field and sufficient time and interest to provide input and feedback on our study. The current Delphi study does not specify the optimal sample size suitable for the study, with the number of experts ranging from three to thirty [31]. If the sample size is too small, it may not be possible to make correct judgments on the issue under discussion, so more experts are needed to obtain more diverse judgments. However, too large a sample size can make it difficult for experts to reach a consensus and the process can become very time-consuming [32]. Since smart health is a relatively new issue, it is not easy to assemble a group of qualified experts for this issue, and the optimal target number of experts for the group is set at 10, which is in line with previous studies [27].

For a multidisciplinary concept such as smart health, the essence of the concept needs to be understood from an integrated perspective by combining the opinions of experts from different backgrounds and fields [33,34,35,36]. We selected professional Delphi experts related to smart health, with all experts stemming from different backgrounds; 10 experts participated in the study, with 2 being from each field (Table 2).

The included participants were as follows: university professors, senior project managers, and information system project managers who had worked in relation to the Internet of Things for more than 20 years; chief doctors and hospital administrators who had worked in China’s first Class III hospitals and had used Internet diagnosis and treatment for more than 15 years; government officials who had worked in the health system for an extensive period, held important positions for more than 15 years, and had rich practical and management experience in the application of medical ICT; and university professors who were deeply engaged in the field of public health and medical health information technology, with more than 15 years of relevant research experience, and were employed as young experts of the Internet Association.

### 2.6. Delphi Rounds

The elements identified after the first round of the survey were listed in an Excel spreadsheet and sent to experts, who were then asked to judge the importance of each element of the “drivers” and “expected outcomes” on a scale from 1 to 5 (see Appendix B). The meanings are given in Table 3.

The indicators related to smart health were determined by assessing the percentage of experts who gave a score of four or five. Based on the literature criteria [37], Table 4 lists the guidelines for selecting indicators, which ensured that the vast majority of respondents agreed with any survey items included. In addition, an opportunity was provided for the project reference group to propose additional indicators. However, only one new indicator was proposed, indicating that most of the expert opinions were already covered in the first round of collection.

All indicators that were considered uncertain in the second round, as well as additional indicators suggested by the project reference group, were sent to the 10 experts in the third round. The scores for each element were made into a table to be sent back to the panelists, and the experts were asked to make a binary choice indicating whether they agreed with the inclusion of each indicator in the framework and provide an explanation for the options constituting disagreement (see Appendix C). The framework incorporated all indicators that were considered mandatory for inclusion by at least 70% of all experts. Subsequently, 13 indicators of consensus were included in the framework.

### 2.7. Consensus and Stability Levels

In accordance with [27], a consensus deviation index (CDI) was used to represent the level of consensus. *r* experts participate in *t* round(s) of the Delphi survey; expert *h* scores item *j* as *X_jht_*. The CDI of the survey results was calculated using the following equation:

$${\mathrm{CDI}}_{\mathrm{jt}}=S_{jt}/\underset{j}{\max}\left\{X_{jt}\right\},\forall j,t$$

where *X_jt_* and *S_jt_* denote the mean and standard deviation of each item, respectively. In addition, a lower CDI indicates that expert opinion is more likely to reach consensus, and when the CDI is zero, expert opinion reaches full agreement. Referring to previous studies, the maximum value of consensus dispersion was set to 0.3 [27,38].

## 3. Results and Analysis

### 3.1. Data Analysis

**Figure 3:**
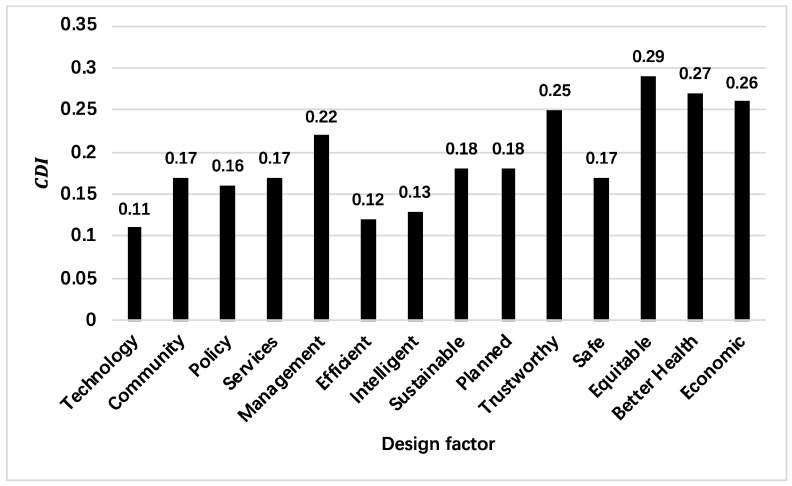
CDI of each factor (the maximum value of consensus dispersion was set to 0.3).

Through the Delphi method, one new factor was added, and two factors were removed, resulting in thirteen factors. Table 5 provides a statistical analysis of the criteria included in each factor. The statistics are the mean, standard deviation, and CDI of the questionnaire results. Figure 3 highlights the differences among the factors in terms of the CDI.

According to the results of the first round of the survey, the CDI of all the survey items was less than 0.3, which shows that the survey results achieved a consensus. According to Table 5, technology, service, and policy received higher average scores and better consensuses in the first round as well as 100% approval in the second round. Community and management, although they did not score well in the first round, passed the second round with 80% and 70% approval rates. The efficient, smart, safe, and economic indicators obtained a higher average score and better consensus in the first round as well as 100% approval in the second round. The sustainable, planned, trustworthy, and equitable indicators, although having performed poorly in the first round, passed the second round with 80% and 70% support, while diverse participation and better health were excluded due to their low support values of 50% and 60%. Ultimately, the following indicators were incorporated into the model: technology, community, policy, services, management, efficient, smart, safe, economic, sustainable, planned, trustworthy, and equitable.

### 3.2. Drivers

#### 3.2.1. Technology

There is no doubt that the development of smart health is driven by technology. No experts questioned the implications of “technology”, but one expert added the perspective of technology acceptance, and we agreed with that expert’s opinion.

The definition of technology can be described on two levels. First, the technology of smart health is still in its infancy in terms of application and has great potential in several areas. Most of the current research on “smart health” is related to the development framework of one or more technologies, which is consistent with the results of the literature review: “No new technology can be carried out in practice without the cooperation of both doctors and patients” (Expert B2).

Previous studies have shown the importance of technology acceptance issues in the health field. First, the perceptions of health workers can greatly influence the use of new technologies [39]. Not only is it difficult for doctors to judge the usability of new technologies, but doctors also feel threatened by ICT due to a loss of authority [40,41] and, subsequently, resist the use of ICT [42]. Secondly, the invasiveness of the technology may cause patients to refuse to try it [43]. The use of monitoring, tracking, and management devices poses ethical challenges for patients, providers, and the social practice of medicine. Patients using the devices may not understand the function of the devices themselves, or may not be able to distinguish between the monitoring and treatment components, resulting in treatment misunderstandings [44,45]. Developers and researchers must, therefore, address this issue in the design and evaluation of new healthcare technologies. However, with the development of technology, there is a greater tolerance and acceptance of smart health [46].

#### 3.2.2. Community

The WHO defines health systems as “all organizations, people and behaviors whose primary purpose is to promote, restore or maintain health” [47]. Thus, a well-developed health system includes not only public hospitals, but also private hospitals, community health facilities, nursing homes, and other types of healthcare organizations and related healthcare workers.

A person-centered health system emphasizes the value of community [48]. The UN Global Strategy has identified community health efforts as one of nine action areas needed to improve health systems [49]. At the same time, however, some experts disagree about the inclusion of “communities” in the smart health framework: “Smart health is still practiced in specific hospitals or specific healthcare scenarios, and less at the community level, although this may be a future trend” (Expert F1).

The role of the community has not been emphasized in most developing-country medical practices, but as experts have noted, it is a “future trend”. The current allocation of healthcare in most developing countries is not rational, having a long history of “emphasizing large hospitals and despising community clinics”, resulting in overconcentration and an uneven distribution of resources. The active participation of smart hospitals in the construction of community health systems can alleviate the problem of an overconcentration of medical resources and the resulting medical crowding [50]. At the same time, many scholars have paid attention to the application of smart health in the elderly care industry with the view of reasonably allocating medical resources and elderly care service resources through smart health so as to organically combine medical care with elderly care [51]. The needs of the elderly regarding high-quality life eliminate the limitations of time and space; consequently, they can enjoy high-quality and comprehensive elderly care services even among their families and communities.

#### 3.2.3. Policy

There are various issues and barriers to the effective implementation of smart health, and policy action is necessary [52]. No experts questioned the “policy” dimension or argued otherwise.

Policies are a must for quality healthcare systems [21]. All smart health practices need to be guided by a well-designed policy framework. Legal, ethical, and social influences are critical to the delivery of health services [53]. Without government supervision, it is impossible to achieve effective coordination for various services across jurisdictions and to share data, information, and knowledge to improve coordination and seamless and comprehensive care. To ensure efficiency in the allocation of public investments, regulatory impact analysis (RIA) and socioeconomic benefit-cost assessments must be conducted so that the needs of stakeholders are met and attention is paid to the expected benefits. At the same time, the government must be involved in building a smart health infrastructure to avoid market failure in this area: “As a new health care technology, smart health may not have enough power to invest in new technology” (Expert E1).

The development, adoption, and dissemination of innovation require the fulfillment of four major factors: metastability, cost, innovation capability, and promotion capability [54]. Not only must smart health compensate the shortcomings of traditional health systems, but it also needs to reduce costs and improve innovation capabilities. In addition, the promotion of leaders is even more important. The innovation tendencies of different regions are highly variable, especially in terms of time preference and risk aversion. Only when innovation does not violate their own interests will stakeholders become the promoters [55]. However, most innovations will show their competitive advantages compared with standard solutions only after a period of time, so it requires great determination on part the of the leaders. If a person is very afraid of taking risks, they will always stick to the current solution. If a person has low time and risk preferences and works in an organization with a coaching and nurturing leadership style, they are likely to become a facilitator.

#### 3.2.4. Service

Smart health services have a significant positive impact on the health of the population [56]. The notion of “service” was never questioned by experts: “Smart health can make it easy and convenient for patients to access care” (Expert C1).

Through the improvement of medical equipment, the optimization of treatment processes, and the ability to diagnose and predict diseases, the application of smart technology has changed the method of value creation in the service system, which has led to the transformation of organizational structures, ecosystems, and innovation models of modern healthcare services [57,58,59]. In the context of smart health, while some small healthcare institutions may not be able to provide certain healthcare services due to limited medical equipment or the skills and capabilities of medical staff, these issues can be overcome with the help of telemedicinal services [60]. The health service represented by “Internet health” can make it easy and convenient for patients to receive medical treatment and to ensure medical fairness.

Some experts also raised concerns about smart health with regard to improving the doctor–patient relationship: “Patients feel comfortable using their phones for monitoring and communicating at all times, but doctors do not. In addition to dissatisfaction with the extra time spent, the validity of the data provided by patients and the description of their condition is also questionable” (Expert B2).

Smart health will change the provider–patient relationship [61]. Trust has always been the pillar of the doctor–patient relationship. The patient believes that the doctor aims to help the patient and maintain confidentiality. In turn, the doctor believes that the patient wants to improve and will follow the prescribed instructions. Theoretically, closer monitoring can lead to better patient outcomes and lower costs. By improving the accuracy of monitoring techniques, high-quality care can be provided, which can also be effective in terms of avoiding conflict in the doctor–patient relationship and improving patient satisfaction.

#### 3.2.5. Management

Many scholars have incorporated the management of workers into traditional healthcare systems [62]. However, the inclusion of “management” in the smart health model is more controversial: “Smart health in practice needs to focus on the clinical practice aspect of the technology, thus achieving progress and breakthroughs in research, and subsequently expanding into areas such as staff management” (Expert C).

We believe that experts only see the “management of people” and ignore the importance of smart health for the management of other healthcare resources. In order to achieve efficient smart health, it is also necessary to use new technologies to improve the “income and expenditure level”, “medical efficiency”, “standard of care”, “waste disposal”, etc., compared to conventional healthcare.

Firstly, smart health has a great demand for people in various fields and should cover all healthcare workers, including doctors, nurses, laboratory staff, etc. [21]. In addition to the number of talents, smart health should also include the broader organizational and environmental factors within the healthcare organization [63]. Secondly, many aspects of traditional financial management need to be changed in order to carry out the further development of smart health. As an important management tool, the advancement of technology provides smart finance [64,65] and smart marketing [66] opportunities and challenges. Effective supply chain management improves operational efficiency and thus reduces costs [67]. Thirdly, with the development of sustainable medicine, medical waste management is gradually becoming the focus of the field’s research [68,69]. More research has focused on reusable medical devices [70], water conservation, waste disposal [71], and energy efficiency [72]. Finally, in a sense, hospitals are public places, and their capacities should be improved so that they can handle various emergencies. Therefore, the construction of a hospital emergency management system is an important foundation to ensure the sustainable development of hospitals [73].

Healthcare management is necessary and is now easily achievable. The development of smart health and the advancement of health technology have all aided health management. Smart health can realize smart management within the hospital from hardware to software and can also provide health services to all people, including healthy people, from the regional to the in-hospital level, from emergency to treatment, and from prehospital to posthospital services.

### 3.3. Outcomes

In addition to improving health, there seems to be growing agreement that the wider goals of health policy include two key economic and social objectives: efficiency and equity. Several countries, such as the USA, the United Kingdom, the Netherlands, Australia, and Canada, have designed and implemented national schemes and indicators with which to measure health system performance, such as efficiency, sustainability, and safety [74]. In addition, different studies have introduced different healthcare goals, such as confidence in the healthcare system [21], achieving a rational allocation of healthcare resources [60], etc. A cross-country comparison, however, requires a comprehensive, international framework such as that of the WHO [75]. The WHO global strategy presents a compelling vision of a future in which people have access to health services that are safe, effective, timely, efficient, and of an acceptable quality [48].

For a description of the different objectives, see Table 1.

#### 3.3.1. Efficient

The shortage of quality healthcare resources is a common problem worldwide, and it is up to healthcare and technology professionals to tap into the “incremental value” of the existing supply and demand environment of healthcare resources and to create efficient solutions through smart health for the benefit of the public.

#### 3.3.2. Smart

Smart health uses AI technology to make digital staff and digital health treatment highly intelligent, thereby partially replacing the medical work previously completed by humans, and constructs a new medical system that integrates the upper and lower levels of gene and disease data at the bottom and diagnosis and surgery at the top; thus, humans and machines are interconnected, cooperative, and advance together.

#### 3.3.3. Security

Smart health care can improve the accuracy of medical testing, and multi-medical platform analysis makes the entire diagnosis and treatment process safe and reliable. At the same time, it renders the operations of medical workers capable of being traced and queried throughout the process. It is also more effective in regulating operations and reducing the occurrence of medical errors and accidents. Regarding the collection of data, the following considerations must be addressed: who has the right to view the data, who has the right to process the data, and how can the respect for the patient’s right to privacy and data control be maximized?

#### 3.3.4. Trust

Trust goes beyond the more traditional notion of satisfaction with care, which is the degree to which people trust and are willing to use healthcare. Trust is critical to maximizing the effectiveness of care because it motivates patients to engage in active participation in their care, such as following the advice of healthcare workers and seeking help from healthcare workers in emergencies.

#### 3.3.5. Economy

By realizing the orderly sharing and mutual recognition of information among medical institutions at all levels, patients can avoid unnecessary duplicate examinations and duplicate dispensation, thereby effectively reducing the burden of medical treatment on the masses. On the other hand, the unified planning and construction of a public health service information system and the unified design and development of application software and various business systems could save a great deal of funds for the overall construction of medical and health information technology.

#### 3.3.6. Sustainability

Smart health is needed to achieve crossovers in multiple fields. The deep integration of the upstream and downstream of the industry chain will aid the sustainable development of the health industry. In addition to research technology enhancement, smart health also needs to be integrated into the economy, society, and environment to meet the important health needs of users.

#### 3.3.7. Planned

In the Internet era, proactive healthcare change is stronger than reactive change. Smart health requires a forward-looking vision and firm will from governments or relevant regulatory bodies to design healthcare reform programs so as to optimize health resource allocation and improve the efficiency of health resource utilization.

#### 3.3.8. Equitable

Governments do not need to render healthcare entirely free; rather, they must make healthcare resources fair and equitable for all. Through smart health, a highly shared, low-infrastructure-requirement, and low-cost healthcare system can be established in a given region, allowing quality healthcare resources to be connected to the majority of the population, thereby achieving equitable distribution at both the medical equipment and medical service levels.

#### 3.3.9. Multiple Participation and Better Health

“This dimension is not required; without this one, it does not affect the advancement of smart health” (Expert E1).

“The medical treatment is more specialized, and it is more difficult and not always effective for patients and other multiple participants” (Expert D1).

“Traditional medicine also has this goal and is not recommended as a result of smart health” (Expert A2).

The experts did not believe these two factors were appropriate for inclusion in a smart health framework. Regarding the participation of multiple agents, since healthcare is led by medical practitioners, new healthcare systems need to be promoted by medical practitioners first and foremost, whereas patients can only passively accept them. Improved health is not a feature of smart health; it does not have enough particularity, so it was excluded.

### 3.4. The Proposed Multidimensional Framework

Currently, many countries or cities around the world are following the smart health trend and declaring that they are implementing or have already implemented smart health. In the context of such rapid urban population growth worldwide, in order to establish a healthcare system that safeguards all citizens, a deeper understanding of smart health that considers both sustainable and balanced development is needed. However, according to this paper, even though smart health is a hot topic with respect to the development of healthcare systems, it is still largely an area that has yet to be studied and practiced, especially from a conceptual point of view. Of course, as the practice of smart health becomes more common, the concept will eventually mature.

**Figure 4:**
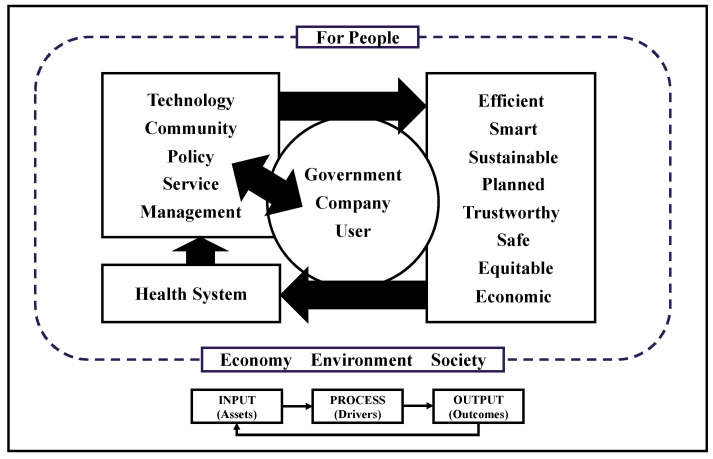
Smart Health Model.

The existing frameworks have some limitations with respect to facilitating our understanding of smart health. Therefore, there is room for the development of new frameworks for smart health. At the conceptual level, in order to establish a thorough understanding—both theoretically and practically—of designing smart health so as to achieve sustainable and balanced growth, we developed a comprehensive framework for smart health, as shown in Figure 4.

In this IPO model, “healthcare” itself—as an “asset”—is an “input”, the five “drivers” (community, technology, policy, services, and management) constitute the “process,” and the “outcome” (efficient, smart, sustainable, planned, trustworthy, participatory, safe, equitable, health-beneficial, and economic) constitutes the “outputs”. Given that the IPO model works effectively and efficiently, the “output” ultimately transforms healthcare (“input”) into smart health.

The 2030 Agenda for Sustainable Development states that a sustainable development system consists of three dimensions, namely, economic, environmental, and social dimensions, which are also part of the “triple bottom line theory” of sustainable development [76]. Thus, three fundamental development areas are placed in the outermost ring of the framework diagram: the economic, social, and environmental dimensions. Describing the cause-and-effect relationship from drivers to smart health development is a complex task; so, it is broken down into individual elements (expected outcomes and sustainability results). The drivers are represented here as opportunities for smart health (new technological developments, policy changes, etc.) and how this translates into achieving the outcomes (e.g., new technologies are used to improve healthcare, governance helps reduce pollution emissions, etc.).

Through the research in this paper, we define smart health as follows:

Smart health puts the patient at the heart of health service design, using smart city infrastructure and technology to integrate fragmented healthcare resources and enable the interaction of patients, providers, medical staff, and medical devices. It can serve as a new way of thinking and living that reimagines care models to enable large-scale, high-quality personalized medicine.

## 4. Discussion

The literature review has revealed that the academic community does not have an excellent definition with which to conceptualize smart health. It is not surprising that the existing definitions still focus on a limited number of areas. This is because the concept of smart health is in its infancy and needs time to accumulate a theoretical and practical basis. In this context, the role of academics is to develop guiding principles and frameworks in order to inform the public and stakeholders about excellent smart health policies and practices. Therefore, this study develops a new multidimensional framework for smart health.

Through the literature review and expert consultation that included relevant people from multiple fields, we have ensured that the drivers of smart health (technology, services, etc.) have not been neglected in the literature so far and have also introduced factors that we consider to be important but neglected (community and management). At the same time, we found relevant studies on the outcomes of smart health with respect to qualities such as economic (efficient), social (equitable), and environmental aspects (sustainable). This study helps enrich the research perspectives of smart health, thus giving full play to the role of medical workers in the construction of smart health, and helps realize the social psychology and cross-fertilization of disciplines, such as public policy and public health.

### 4.1. Smart Health and Relevant Concept

Smart healthcare is a higher stage of information construction in the medical field [10]. Distinct from health informatization and digital health, smart health is the provision of health services by using the context-aware network and sensing infrastructure of smart cities, which promotes the interaction between all parties in the healthcare field, ensures that participants receive the services they need, helps the involved parties make informed decisions, and facilitates the rational allocation of resources.

The terms smart health, electronic health (e-health), or Internet health are often misused in research. According to the conclusion of this article, smart health emphasizes a situation and a method of thinking and living. E-health has plenty of definitions, but whether electronic health records (EHR) or databases are used to store the medical information of patients [77], it is a path or tool to achieve the expected state. As a sub-segment of e-health [78], mobile health—or telemedicine—is also used to achieve the goal of smart health.

### 4.2. Smart Health and Smart City

Smart health is often considered as an application of smart cities [79] and its successful implementation requires the use of the infrastructure provided by the smart city; thus, healthcare is a long-standing issue in smart cities. In recognition of this trend, scholars have highlighted the potential of smart cities to enhance the health and well-being of their citizens [80].

As a new path to achieve sustainable urban development in countries around the world, smart cities have consumed vast sums of money over the past decade, but problems such as the disconnect between smart health and smart city construction have hampered the achievement of said urban development’s due effectiveness with regard to responding to new health needs. The integration and development of smart health and smart cities will be crucial for future smart cities to be able to build a modern public health management system and form truly sustainable competitiveness in the city. We hereby advocate that smart health and related public health systems should be applied to a topic that has received widespread attention from scholars and practitioners alike in recent years: smart cities.

### 4.3. Delphi Methodology for Identifying Smart Health Factors

The research method of this study provides researchers with a new approach to health system development. In the academic research related to health systems, few studies use the Delphi method. The Delphi method is widely used in different academic fields to obtain appropriate consistency among experts. In this study, one of the advantages of the Delphi method is that the factors of smart health are derived from the opinions of different people, whose skills are most closely related to smart health rather than a specific field. Smart health issues are not only related to specific users in a hospital but to multiple stakeholders as well. Stakeholder engagement in smart health is groundbreaking because of its impact on health service quality and public health [81]. Therefore, it is important to consider the different stakeholders’ needs and interests at all stages of health service delivery. Such engagement can lead to higher stakeholder satisfaction and increase the likelihood of service success [82]. Stakeholders are individuals or groups who can influence or are influenced by the actions of entities such as organizations, programs, or even services, such as patients, families, legislators, doctors, companies that provide healthcare services, technology companies, etc.

### 4.4. Implications for Practice

With the increasing public demand for medical care and the consequent changes in medical services, the discovery of a method for reasonably allocating medical resources according to different conditions is also a major problem faced by smart health, which requires effective community services. Smart health needs the foundation of a health information system. In some developed areas, mainly big cities, HIS has been established, but in rural areas, the development speed is much slower. In the coming years, more resources should be invested in these areas to narrow the gap. Investments should include not only hardware platforms, necessary equipment, and software systems, but also human resources. Efforts should be made to encourage more medical personnel and experienced technicians to relocate to rural areas and communities. We found that the development of smart health necessitates the consideration of the construction of community medical care and tertiary medical institutions. Large hospitals, small hospitals, and community hospitals will be combined through informatization so that hospital experts can promptly help community service centers and enhance the level of disease diagnosis and treatment at the grass-roots level.

Technology is not the main problem at this stage. Solving social and economic problems is the first step for the sustainable development of smart health. At present, neither doctors nor patients have enough interest or ability to bravely take the first step in the implementation of smart health. It is extremely important to improve smart health literacy. From the perspective of patients, smart health lacks relevant legal norms, which poses risks of personal information and privacy disclosure. Meanwhile, patient satisfaction is crucial to the sustainable development of smart health, which also reflects the patient-centered principle of smart health. This needs to be achieved through advanced technology, high-quality service, and good management.

Technology companies are also very important to the development of smart health. In fact, they can play a leading role in the development of smart health technology. They are the architects of the final information platform. They know what customers need and where their weaknesses are. However, few companies are willing to take risks in basic research because there are not enough resources. In this case, the government should support some selected companies in the performance of basic research and lead the development of smart health.

### 4.5. Limitations

When interpreting the specific findings of the study, however, the reader must be aware of the following limitations: (1) Our search for peer-reviewed full-text articles may have overlooked conference proceedings, book chapters, and white papers. (2) The unconscious bias of the authors may have influenced the execution of the review and the interpretation of the findings. (3) The panelists were all from China; thus, our findings and framework may need to be adapted to other populations and countries.

### 4.6. Future Direction

Future work could develop a comprehensive smart health scale or evaluation index to better evaluate the development level of smart health in a country or region after defining the concept of smart health. Moreover, future work could target specific criteria. Other studies could make use of the analytical hierarchy process (AHP) to weigh the criteria and build a framework that is usable in a practical situation. For doctors and patients, future research can involve the impact of smart health on behavior and psychology. For the government, we can form several policy recommendations that will help develop smart health and improve the service quality of medical personnel; additionally, we can carry out field experiments and investigation experiments to test these aspects.

## 5. Conclusions

This paper proposes a comprehensive framework for smart health that links the drivers and outcomes of smart health. We have ensured that the drivers of smart health (technology, services, etc.) have not been neglected in the literature so far and have also introduced factors that we consider important but neglected (community and management). In addition, we briefly described the differences between smart health and related concepts and concluded that the infrastructure of smart cities is indispensable for the development of smart health. We combined a systematic, critical review of the interdisciplinary literature on smart health and modified the Delphi method; moreover, we invited experts to participate in group discussions in order to reduce the occurrence of omissions in our literature review. By employing the proposed framework, future research can enrich the smart health scale or evaluation indicators, explore the impact path of smart health on relevant practitioners through empirical research, and further demonstrate how each subject should be implemented through a balanced, sustainably developed approach.

## Figures and Tables

**Table 1:** Smart health factors and definition.

Design Factor	Definition
Technology	Smart health is based on information and communication technology (ICT) and is used to connect hospital staff, data, devices, core systems, and core infrastructure through the Internet of Things (IoT) for better diagnosis and treatment. Smart health also requires the acceptance of new technologies by both doctors and patients.
Service	With the help of an efficient medical system, smart medical care can streamline the process of medical treatment, improve the efficiency of medical treatment, facilitate communication between doctors and patients, and realize paperless and standardized case management through “Internet medicine”, “telemedicine”, and “consultation navigation”. It also enables paperless and standardized case management.
Policy	Achieving the goal of smart health requires government leadership within a well-designed framework. It includes a series of regulations and actions, such as rational allocation of healthcare resources, provision of basic health insurance, a configuration of the healthcare infrastructure, and multisectoral coordination.
Community	Through community medical service centers, smart medical care establishes electronic health records for community residents, tracks the health of community residents (especially the elderly), provides basic medical services, establishes (mandatory) referral systems, and reduces medical pressure on large hospitals.
Management	Smart health requires a systematic division of labor among several specialties and a rational allocation of manpower (recruitment, revenue allocation, etc.), finance (assets, prices, etc.), and safety (graded care, pollutant discharge, etc.) through medical (hospital) information systems.
Efficient	Smart medicine improves the efficiency of staff and patient access through system optimization, thereby increasing the satisfaction of both doctors and patients.
Intelligent	Smart health combines the concepts of evidence-based medicine and specialized treatment with connected platforms and data to provide fast and accurate access to treatment options.
Sustainable	Smart health establishes a new management model, attracts talent, and promotes knowledge upgradation as well as the patient-centered and robust development of the medical insurance system.
Planned	The government provides the framework for smart health development, leads proactive change in the healthcare industry, fulfills regulatory and leadership obligations, and harmonizes healthcare data standards.
Trustworthy	Compared to traditional healthcare, the smart health model can improve patient satisfaction, build trust between doctors and patients, and reduce doctor–patient conflicts.
Equitable	Smart health reduces the risk of medical errors and substandard care, properly manages medical data, and maintains patient privacy. It demonstrates good resilience in the event of crises, such as medical cramming and paralysis.
Fair	Smart health achieves medical coverage for all by rationally allocating medical resources, avoiding excessive concentration of resources, and narrowing the gap between urban and rural areas.
Better Health	Smart health can increase the average life expectancy of society, reduce disease morbidity and mortality, reduce disease suffering, and improve quality of life.
Economic	Smart health leverages smart technology to reduce healthcare costs while reducing ineffective and harmful healthcare waste through community-based hierarchical care.

**Table 2:** Demographics of the participants.

Domain	Code	Time (Year)	Career	Degree
Intelligent Technologist	A1	22	University Professor	PhD
	A2	7	Corporate R&D staff	Undergraduate
Doctors	B1	19	Neurologist	PhD
	B2	5	Neurologist	PhD
Hospital Administrators	C1	10	Medical Service	Master’s
	C2	12	Medical Service	Master’s
Government Officials	D1	17	Government Officials	Undergraduate
	D2	16	Government Officials	Undergraduate
Research Scholars	E1	19	University Professor	PhD
	E2	17	University Professor	PhD

**Table 3:** Rating scale specifying the perceived impacts of smart health indictors.

Rating Scale	Perceived Impact
1	no impact
2	small impact
3	moderate impact
4	large impact
5	very high or profound impact

**Table 4:** Guidelines governing inclusion of indicators in the smart health framework.

Percentage of Experts Indicating a Large or Profound Impact	Decision
70% and above	Include
40% to 69%	Indeterminate
39% and below	Exclude

**Table 5:** Criteria consensus.

Design Factor		First Round				Second Round
		Mean	SD	CDI_jt_	Number of Consenting Participants (N = 10)	Number of Consenting Participants (N = 10)
Drivers	Technology	4.3	0.50	0.11	10	/
	Community	3.3	0.78	0.17	4	8
	Policy	4.5	0.73	0.16	9	/
	Services	4.1	0.78	0.17	8	/
	Management	3.5	1.01	0.22	6	7
Outcomes	Efficient	4.6	0.53	0.12	10	/
	Intelligent	4.1	0.60	0.13	9	/
	Sustainable	3.7	0.83	0.18	7	/
	Planned	3.7	0.83	0.18	5	7
	Trustworthy	3.6	1.12	0.25	7	/
	Safe	4.1	0.78	0.17	8	/
	Equitable	3.8	1.32	0.29	6	8
	Better Health	3.5	1.22	0.27	6	6
	Economic	3.8	1.20	0.26	8	/
	Multiple Participation	New				5

## Data Availability

Data sharing not applicable.

## Appendix A. Definition and Primary Theme of Smart Health

**NO**	**Reference**	**Definition**	**Theme**
1	[83]	A healthcare system that enables patients and doctors to communicate with each other and remotely exchange the information monitored, collected, and analyzed from patients’ daily activities via the IoT.	Technology Services Efficiency
2	[18]	Smart healthcare can be defined as an integration of patients and doctors into a common platform for intelligent health monitoring by analyzing day-to-day human activities.	Technology Services
3	[10]	Smart healthcare uses a new generation of information technologies, such as the internet of things (loT), big data, cloud computing, and artificial intelligence, to transform the traditional medical system in an overarching fashion, thereby rendering healthcare more efficient, convenient, and personalized.	Technology Efficient Trust Sustainable
4	[84]	Smart Health provides the healthiest possible living environment by improving quality of life. Combining disruptive technologies (Internet of Things (IoT) + Cloud Computing + Smart Sensing + Big Data technologies), this system constitutes a paradigm shift in the field of ICT that seeks to promote and render optimal solutions and care coordination in a form of collaborative management called “smart health”.	Technology Services Management Better -health Efficiency
5	[85]	The emerging field of s-health constitutes isolated, intelligent, customized health services, usually employing sensor data gathering and cloud processing.	Technology Services
6	[77]	Smart health is the provision of health services by using a context-aware network and the sensing infrastructure of smart cities.	Technology Services
7	[86]	This term, which inherently integrates ideas from ubiquitous computing and ambient intelligence applied to the future P4-medicine concept, is tightly connected to concepts of wellness and well-being, and incorporates big data, collected by vast quantities of biomedical sensors and actuators, to monitor, predict, and improve patients’ physical and mental conditions.	Technology Efficiency Health
8	[60]	Intelligent medicine refers to the construction of an interactive platform for the sharing of medical information based on electronic health records and the comprehensive use of the IoT, internet, cloud computing, big data, and other technologies to realize the interaction of patients, medical institutions, and medical personnel and equipment, and intelligently match the needs of the medical biosphere.	Technology Services Efficiency Sustainable
9	[8]	The infrastructure and technology of smart cities reconstruct the thinking behind existing healthcare systems (e.g., m-health, e-health, etc.) and telemedicine to create a new and comfortable ubiquitous concept that is called smart health.	Technology Thinking
10	[87]	Smart health integrates ideas from ubiquitous computing and ambient intelligence applied to predictive, personalized, preventive, and participatory healthcare systems.	Technology Efficiency Health Trust Sustainable
12	[88]	Smart health refers not only to ICT development, but also to a state of thinking, a lifestyle and approach, and a vow for connected entities to improve healthcare facilities in the home, city, country, and globe with the aid of a number of intelligent agents.	Technology Services Thinking Efficiency

## Appendix B. MDM Questionnaire (Round 2)

- Rules for filling in the survey: please give a score according to your understanding of the importance of the drivers (projects) of smart healthcare. (Round 2.).

**Design Factor**	**Please Circle One Number Per Row below Using the Scale:**				
	**1 Being Very Unimportant and 5 Being Very Important**				
Technology	1	2	3	4	5
Service	1	2	3	4	5
Policy	1	2	3	4	5
Community	1	2	3	4	5
Management	1	2	3	4	5
Other influencing factors (if any):					

B. Rules for filling in the survey: please give a score according to your understanding of the importance of the outcomes (target) of smart healthcare. (Round 2).

**Design Factor**	**Please Circle One Number Per Row below Using the Scale:**				
	**1 Being Very Unimportant and 5 Being Very Important**				
Efficient	1	2	3	4	5
Intelligent	1	2	3	4	5
Sustainable	1	2	3	4	5
Planned	1	2	3	4	5
Trustworthy	1	2	3	4	5
Safe	1	2	3	4	5
Equitable	1	2	3	4	5
Better Health	1	2	3	4	5
Economic	1	2	3	4	5
Other influencing factors (if any):					

## Appendix C. MDM Questionnaire (Round 3)

Please indicate if you approve of the drivers (projects) or outcomes (target) below. If you do not approve of any of the design factors, please indicate why in the space provided after the statements. (Round 3)

**Design Factor**		**Approve/** **Do Not Approve**	**Reason**
Drivers	Community		
	Management		
Outcomes	Planned		
	Equitable		
	Better Health		
	Multiple participation		
